# The Reference Site Collaborative Network of the European Innovation Partnership on Active and Healthy Ageing

**Published:** 2019-01-06

**Authors:** J Bousquet, M Illario, J Farrell, N Batey, AM Carriazo, J Malva, J Hajjam, E Colgan, N Guldemond, M Perälä-Heape, GL Onorato, A Bedbrook, L Leonardini, V Stroetman, S Birov, C Abreu, A Abrunhosa, A Agrimi, T Alalääkkölä, N Allegretti, F Alonso-Trujillo, M Álvarez-Benito, S Angioli, J Apóstolo, G Armitage, S Arnavielhe, M Baena-ParejoI, PD Bamidis, A Balenović, M Barbolini, I Baroni, H Blain, PL Bernard, M Bersani, E Berti, L Bogatyrchuk, R Bourret, J Brehm, L Brussino, D Buhr, D Bultje, E Cabeza, A Cano, C De Capitani, E Carantoña, A Cardoso, JI Coll Clavero, B Combe, D Conforti, L Coppola, F Corti, E Coscioni, E Costa, G Crooks, A Cunha, C Daien, Dantas, J Darpón Sierra, M Davoli, A Dedeu Baraldes, V De Luca, L De Nardi, M Di Ciano, A Dozet, B Ekinci, S Erve, JM Espinoza Almendro, A Fait, R Fensli, S Fernandez Nocelo, P Gálvez-Daza, J Gámez-Payá, M García Sáez, I Garcia Sanchez, B Gemicioğlu, W Goetzke, E Goossens, M Geurdens, Z Gütter, H Hansen, S Hartman, G Hegendörfer, H Heikka, D Henderson, D Héran, S Hirvonen, G Iaccarino, N Jansson, H Kallasvaara, F Kalyoncu, U Kirchmayer, JA Kokko, J Korpelainen, T Kostka, P Kuna, T Lajarín Ortega, CM Lama, D Laune, D Lauri, V Ledroit, G Levato, L Lewis, G Liotta, L Lundgren, F Lupiañez-Villanueva, P Mc Garry, M Maggio, E Manuel de Keenoy, C Martinez, M Martínez-Domene, B Martínez-Lozano Aranaga, M Massimilliano, A Maurizio, O Mayora, C Melle, A Mendez-Zorilla, H Mengon, G Mercier, J Mercier, I Meyer, A Millet Pi-Figueras, P Mitsias, DW Molloy, R Monti, ML Moro, H Muranko, M Nalin, A Nobili, M Noguès, R O’Caoimh, S Pais, D Papini, P Parkkila, C Pattichis, A Pavlickova, A Peiponen, S Pereira, JL Pépin, J Piera Jiménez, P Portheine, L Potel, AC Pozzi, P Quiñonez, X Ramirez Lauritsen, MJ Ramos, A Rännäli-Kontturi, A Risino, C Robalo-Cordeiro, G Rolla, R Roller, M Romano, V Romano, J Ruiz-Fernández, C Saccavini, A Sachinopoulou, MJ Sánchez Rubio, L Santos, S Scalvini, E Scopetani, D Smedberg, R Solana-Lara, B Sołtysik, M Sorlini, S Stericker, M Stramba Badiale, I Taillieu, M Tervahauta, A Teixeira, H Tikanmäki, A Todo-Bom, A Tooley, A Tuulonen, C Tziraki, S Ussai, S Van der Veen, A Venchiarutti, D Verdoy-Berastegi, M Verissimo, L Visconti, M Vollenbroek-Hutten, K Weinzerl, L Wozniak, A Yorgancıoğlu, V Zavagli, AJ Zurkuhlen

**Affiliations:** 1 MACVIA-France, Fondation partenariale FMC VIA-LR, Montpellier, France; 2 VIMA, INSERM U 1168, VIMA : Ageing and chronic diseases. Epidemiological and public health approaches, Villejuif, Université Versailles St-Quentin-en-Yvelines, UMR-S 1168, Montigny le Bretonneux, France, Euforea, Brussels, Belgium, and Charité, Universitätsmedizin Berlin, Humboldt-Universität zu Berlin, and Berlin Institute of Health, Comprehensive Allergy Center, Department of Dermatology and Allergy, Berlin, Germany; 3 Division for Health Innovation, Campania Region and Federico II University Hospital Naples (R&D and DISMET) Naples, Italy; 4 LANUA International Healthcare Consultancy, Down, UK; 5 EIPonAHA Reference Site Collaborative network, Head of EU & International Funding, Health and Social Services Group, Welsh Government, Cardiff, UK; 6 Regional Ministry of Health of Andalusia, Seville, Spain; 7 Institute of Biomedical Imaging and Life Sciences (IBILI), Faculty of Medicine, University of Coimbra; Coimbra, and Ageing@Coimbra EIP-AHA Reference Site, Coimbra, Portugal; 8 CENTICH Mutualité Française Anjou Mayenne, Angers, France; 9 Department of Health, Social Services and Public Safety, Northern Ireland Belfast, UK; 10 Institute of Health Policy and Management iBMG, Erasmus University, Rotterdam, The Netherlands; 11 Oulu University, Faculty of Medicine, Oulu, Finland; 12 Veneto Region, Mattone Internazionale Program, Italy; 13 Empirica Communication and Technology Research, Bonn, Germany; 14 Nursing School of Coimbra, Ageing@Coimbra, Coimbra, Portugal; 15 Comissão de Coordenação e Desenvolvimento Regional do Centro (CCDRC), Ageing@Coimbra EIP-AHA Reference Site, Coimbra, Portugal; 16 Aprulia Region - Research, Innovation and Capacity Building department, Bari – Italy; 17 Oulu Health Labs, Oulu, Finland; 18 LISPA, Milano, Italy; 19 Agency for Social Services and Dependency of Andalusia, Seville, Spain; 20 Campania Councillor for European Funds, Euromediterranean Basin and Youth Policies, Naples, Italy; 21 Newcastle University, Operations Director, National Innovation Centre for Ageing, New Castle, UK; 22 Kyomed, Montpellier France; 23 Medical Education Informatics; Lab of Medical Physics; Medical School; Aristotle University of Thessaloniki, Greece; 24 Health Care Center Zagreb, City of Zagreb, AHA Reference site, Zagreb, Croatia; 25 Regione Emilia Romagna - Agenzia Sanitaria e Sociale, Regional Health and Social Agency Emilia-Romagna, Reference Site of the European Innovation Partnership on Healthy and Active Ageing, Bologna, Italy, and EU Commission Senior Public Health Expert; 26 Telbios SRL, Milan, Italy; 27 Department of Geriatrics, Montpellier University Hospital, Montpellier, France; 28 EUROMOV. EA 2991, Euromov, University of Montpellier, France; 29 Sport Faculty, University of Montpellier, France; 30 Head Unit Plans and Projects; DG Welfare – Region of Lombardy, Milano (Italy); 31 Regional Health and Social Agency Emilia-Romagna, Bologna, Italy; 32 The medical improving center “Elbrus”, Zhytomir, Ukraine; 33 Centre Hospitalier Valenciennes, France; 34 Health region CologneBonn, Köln, Germany; 35 Department of Medical Sciences, Allergy and Clinical Immunology Unit, University of Torino & Mauriziano Hospital, Torino, Italy; 36 University of Tuebingen / Steinbeis Transfercenter for Social and Technological Innovation, Tuebingen, Germany; 37 Healthy Ageing Network Northern Netherlands, Groningen, The Netherlands; 38 Cap de Servei de Promoció de la Salut, Direcció General de Salut Pública i Participació, Palma de Mallorca, Spain; 39 Department of Pediatrics, Obstetrics and Gynecology, University of Valencia, Spain; 40 INCLIVA, Valencia, Spain; 41 Lombardy Cluster Technologies for Living Environments, Lecco (LC), Italy; 42 Consejería de Presidencia y Participación Ciudadana, Oviedo, Spain; 43 Innovation and new technologies, Hospital de Barbastro Servicio Aragones de Salud Aragon, Spain; 44 Department of Rheumotology, University Hospital, Montpellier, France; 45 Autonomous Province of Trento, Health and Social Solidarity Department & TrentinoSalute4.0, Trento, Italy; 46 Head Unit Health Promotion and Screening; DG Welfare – Region of Lombardy, Milan, Italy; 47 FIMMG, Federazione Italiana Medici di Medicina Generale, Milan, Italy; 48 Department of Heart Surgery, San Giovanni di Dio e Ruggi d’Aragona Hospital, Salerno, Italy; 49 UCIBIO, REQUIMTE, Faculty of Pharmacy of University of Porto, Porto4ageing Reference Site, University of Porto, PORTO, Portugal; 50 Scottish Centre for Telehealth and Telecare, NHS 24, Glasgow, UK; 51 Instituto Pedro Nunes, Ageing@Coimbra EIP-AHA Reference Site, Coimbra, Portugal; 52 Cáritas Diocesana de Coimbra, Ageing@Coimbra EIP-AHA Reference Site, Coimbra, Portugal; 53 Regional Minister of Health, Basque Region, Bilbao, Spain; 54 Department of Epidemiology, ASL Roma 1, Lazio Regional Health Service, Roma, Italy; 55 Agency for Health Quality & Assessment of Catalonia of the Ministry of Health of Catalonia – AquAs, Barcelona, Spain; 56 R&D Unit, Federico II University Hospital, Naples, Italy; 57 Health Information System International Projects, Lombardia Informatica SpA, Milano, Italy; 58 InnovaPuglia - Inhouse ICT company of Regione Puglia and Reference Site Puglia WI-FI Management, Bari, Italy; 59 Health economist, Region Skåne, Sweden; 60 Head Chronic Disease Department, Ministry of Health, Ankara, Turkey; 61 Health and Social Care Directorate, ATS Città Metropolitana (Health and Social Care Agency), Milano, Italy; 62 Centre of eHealth and Health Care Technology, University of Agder, Faculty of Engineering and Science, Grimstad, Norway; 63 Galician Health Knowledge Agency (ACIS), Regional Ministry of Public Health of Galicia; 64 Regional Ministry of Equality and Social Policies of Andalusia, Seville, Spain; 65 ADVANTAGE JA, SERMAS Hospital de Getafe, Spain; 66 Department of Pulmonary Diseases, Istanbul University-Cerrahpasa, Cerrahpasa Faculty of Medicine, Istanbul, Turkey; 67 Center for Gastrology, School of Gastrologic Sciences and Primary Food Care, Leuven, Belgium; 68 Center of Expertise in Primary Food Care, Center for Research and Innovation in Care (CRIC), Antwerp, Belgium; 69 University Hospital Olomouc - NTMC, National eHealth Centre, Olomouc, Czech Republic; 70 EU Consultant & Project Manager, South Denmark European Office, Brussels, Belgium; 71 Department of Social Services and Health Care, Business Development, HELSINGIN KAUPUNKI, City of Helsinki, Finland; 72 Saxony Liaison Office Brussels, Brussels, Belgium; 73 Head of European Engagement, NHS 24, Glasgow, UK; 74 CARSAT, Montpellier, France; 75 City of Oulu, Oulu, Finland; 76 Department of Advanced Biomedical Sciences, University of Naples Federico II, Naples, Italy; 77 Network Ecosystem, BusinessOulu, Oulu, Finland; 78 Helsinki-Uusimaa Regional Council, Helsinki, Finland; 79 Hacettepe University, School of Medicine, Department of Chest Diseases, Immunology and Allergy Division, Ankara, Turkey; 80 Department of Healthcare and Social Welfare, Technology Specialist, Oulu, Finland; 81 Oulu University Hospital OYS, Hospital District, Oulu, Finland; 82 Department of Geriatrics, Medical University of Lodz, Healthy Ageing Research Centre (HARC), Lodz, Poland; 83 Division of Internal Medicine, Asthma and Allergy, Barlicki University Hospital, Medical University of Lodz, Poland; 84 Committee of Representatives of People with disabilities and their Families, Region de Murcia, Spain; 85 CMMC, Milano, Italy; 86 Alsace Lorraine Champagne Ardenne, Bureau Europe Grand Est, Bruxelles, Belgique; 87 SIFMED, Scuola Italiana Di Formazione E Ricerca In Medicina Di Famiglia, Milan, Italy; 88 Head of Research and Development, International Foundation for Integrated Care and EIP on AHA B3 Action Group Chair, Wolfson College, Oxford, UK; 89 Biomedicine and Prevention Department, University of Rome Tor Vergata, Rome, Italy; 90 Development Department, Region Norrbotten, Sweden; 91 Information and Communication Sciences, Universitat Oberta de Catalunya, Spain; 92 Greater Manchester Ageing Hub, Greater Manchester Combined Authority, Manchester, UK; 93 Department of Medicine and Surgery - Geriatric Clinic Unit Department of Medicine Geriatric Rehabilitation, University Hospital of Parma, Italy; 94 Kronikgune, International Centre of Excellence in Chronicity Research, Barakaldo, Bizkaia, Spain; 95 Costa Cálida Cares-Senior Tourism and Services, Region de Murcia, Spain; 96 Regional Health Ministry, Region de Murcia, Spain; 97 Financial Range for Innovation, Research, International care and health sector; Friuli Venezia Giulia Autonomous Region, Central Directorate for Health, Social Health Integration, Social Policies and Family, Trieste, Italy; 98 Plans and Projects Unit, DG Welfare – Region of Lombardy, Italy; 99 Bruno Kessler Foundation, eHealth Unit and TrentinoSalute4.0, Trento, Italy; 100 Care Management Unit, Hausach, Gesundes Kinzigtal GmbH, Kizingtal, Germany; 101 University of Deusto, Bilbao, Spain; 102 Unité Médico-Economie, Département de l’Information Médicale, University Hospital, Montpellier, France; 103 Department of Physiology, CHRU, University Montpellier, PhyMedExp, INSERM U1046, CNRS UMR 9214, France; 104 Centre de Vida Independent CVI, Barcelona, Spain; 105 Department of Neurology, School of Medicine, University of Crete, Heraklion, Crete, Greece; 106 Centre for Gerontology and Rehabilitation, School of Medicine, UCC @ St Finbarr’s Hospital, Cork, Ireland; 107 GEWI Institute, Regional Innovation Partnership on Active and Healthy Ageing, Köln, Germany; 108 Mario Negri Institute for Pharmacological Research, IRCCS; Clinical Pharmacology, Geriatrics, Internal Medicine, Milano, Italy; 109 Palavas, Occitanie, France; 110 Health Research Board, Clinical Research Facility Galway, National University of Ireland, Galway, Ireland; 111 Center for Biomedical Research-CBMR, Department of Biomedical Sciences and Medicine, International Center on Ageing-CENIE, University of Algarve, Portugal; 112 Dept of Computer Science, University of Cyprus, Cyprus, Greece; 113 European Service Development Manager, NHS 24, Glasgow, UK; 114 Social services and health care division, Hospital, rehabilitation and care services, Southern service district, City of Helsinki, FINLAND; 115 University of Porto and Porto4Ageing Reference Site, Porto, Portugal; 116 Université Grenoble Alpes, Laboratoire HP2, Grenoble, INSERM, U1042 and CHU de Grenoble, France; 117 Information and R&D Officer, Badalona Serveis Assistencials, Badalona, Spain; 118 Coöperatie Slimmer Leven, Eindhoven, The Netherlands; 119 International Affairs & Public Procurement of Innovation, Hospital Procurement Network, Paris, France; 120 IML, Lombardy Medical Initiative, Bergamo, Italy; 121 ZealandDenmark, EU Health and Care affaires, Copenhagen, Denmark; 122 UCIBIO, REQUIMTE, Faculty of Sciences of University of Porto and Porto4Ageing Reference Site, Porto, Portugal; 123 International Affairs, OuluHealth, Oulu, Finland; 124 Health Innovation Manchester, Manchester, UK; 125 Faculty of Medicine, University of Coimbra, Portugal, Ageing@Coimbra EIP-AHA Reference Site; 126 Medical University of Graz, Department of Internal Medicine, Graz, Austria; 127 IRES - Institute for Economic and Social Research - Piedmont, Torino, Italy; 128 Arsenàl.IT, Veneto’s Research Centre for eHealth Innovation, Venice, Italy; 129 Oulu University, Center of Health and Technology, Oulu, Finland; 130 Odem dos Farmacêuticos, Secção Regional do Centro, Ageing@Coimbra EIP-AHA Reference Site, Coimbra, Portugal; 131 Cardiology Rehabilitation Division, Salvatore Maugeri Foundation IRCCS, Institute of Lumezzane, Brescia, Italy; 132 Tuscany Region, Directorate Citizenship rights and social cohesion, Firenze, Italy; 133 RISE Research Institutes of Sweden, Division Safety and Transport - Measurement Science and Technology, Lund, Sweden; 134 Head of Programmes, Yorkshire & Humber Academic Health Science Network, Wakefield, UK; 135 Department of Geriatrics and Cardiovascular Medicine, IRCCS Istituto Auxologico Italiano, Milano, Italy; 136 Coördinator Zorgeconomie, Fabrieken voor de Toekomst, Brugge, Belgium; 137 Social and Health Services, Kuopio, Finland; 138 Faculty of Sport Sciences and Physical Education, University of Coimbra, Ageing@Coimbra EIP-AHA Reference Site, Portugal; 139 Life Science Industries and Company Networks, BusinessOulu, Oulu, Finland; 140 Tays Eye Centre, Tampere University Hospital, Pirkanmaa Hospital District, Tampere, Finland; 141 Research and Evaluation Department, Municipality of Jérusalem, Israël; 142 Medicine and Health Care Science, Allilegi Community Based Organization for AD and Active Healthy Aging, Heraklion, Crete, Heraklion-Crete Reference Site Region, Greece; 143 DG Welfare, Lombardy Region, Italy; 144 Department of Med Hum, Amsterdam University Medical Centers, VU University, NL; 145 Friuli Venezia Giulia Autonomous Region, Central Directorate for Health, Social Health Integration, Social Policies and Family, Trieste, Italy; 146 LifeTechValley, Life Sciences Incubator BioVille, Diepenbeek, Belgium; 147 University of Twente, Biomedical systems and signal group/telemedicine, Twente, The Netherlands; 148 Human.technology Styria GmbH, Graz, Austria; 149 Research and International Relations, Department of Structural Biology, Medical University of Lodz, Lodz, Poland; 150 Celal Bayar University, School of Medicine, Department of Pulmonology, Manisa, Turkey; 151 ANT Foundation, Bologna, Italy

**Keywords:** Active and healthy ageing, European Innovation Partnership on Active and Healthy Ageing, EIP on AHA, DG CONNECT, DG Santé

## Abstract

Seventy four Reference Sites of the European Innovation Partnership on Active and Healthy Ageing (EIP on AHA) have been recognised by the European Commission in 2016 for their commitment to excellence in investing and scaling up innovative solutions for active and healthy ageing. The Reference Site Collaborative Network (RSCN) brings together the EIP on AHA Reference Sites awarded by the European Commission, and Candidate Reference Sites into a single forum. The overarching goals are to promote cooperation, share and transfer good practice and solutions in the development and scaling up of health and care strategies, policies and service delivery models, while at the same time supporting the action groups in their work. The RSCN aspires to be recognized by the EU Commission as the principal forum and authority representing all EIP on AHA Reference Sites. The RSCN will contribute to achieve the goals of the EIP on AHA by improving health and care outcomes for citizens across Europe, and the development of sustainable economic growth and the creation of jobs.

## I. INTRODUCTION

As populations continue to grow older it is important to support the process of ageing well, active and healthy, that is a priority objective ^[1]^. The broad concept of active and healthy ageing (AHA) is the process of optimizing opportunities for health and social care to increase healthy life expectancy, healthy life years and quality of life for all people as they age ^[2,3]^. AHA allows people to realize their potential for physical, social and mental wellbeing throughout the life course ^[4]^. AHA also contributes to the sustainability of our health and social systems, reducing dependency and disability.

To tackle the potential and challenges of ageing in the EU, the EC - within its Innovation Union policy-launched the European Innovation Partnership on Active and Healthy Ageing (EIP on AHA) ^[5,6]^ in 2012. It continues to pursue a triple win for Europe (https://webgate.ec.europa.eu/eipaha/):

- Enabling EU citizens to lead healthy, active and independent lives while ageing.
- Improving the sustainability and efficiency of social and health care systems.
- Boosting and improving the competitiveness of the markets for innovative products and services, responding to the ageing challenge..

The EIP on AHA brings together public and private stakeholders to accelerate the deployment of major innovations by committing them to undertaking supply and demand side measures across sectors and the entire innovation chain. EIP on AHA does not replace existing decision-making processes. However, regional commitments to EIP on AHA can influence and inform policy decisions, support change management strategies and service delivery models, and therefore identify opportunities, and potential partners, under a range of funding programmes for the development of evidence based innovative solutions.

The EIP on AHA is a distinctive opportunity to help deliver on the policy objectives of the Europe 2020 flagships. Its objectives and approach are also in line with the principles and goals of the EU Health Strategy “Together for Health”.

## II. REFERENCE SITES OF THE EUROPEAN INNOVATION PARTNERSHIP ON ACTIVE AND HEALTHY AGEING

Reference Sites (RS) are “ecosystems which comprise various players (including regional and/or local government authorities, cities, hospitals/care organisations, industry, SMEs and/or start-ups, research and innovation organisations including universities and civil society), that jointly implement a comprehensive, innovation-based approach to AHA, and can give evidence and concrete illustrations of the impact of such approaches on the ground” (https://ec.europa.eu/eip/ageing/reference-sites_en).

In 2012, 32 RS were awarded by the EU. Networking is a fundamental part of the EIP on AHA. RSs are pioneering together some of the most advanced innovative solutions to improve the lives of its ageing populations and through the partnership. Through “maturity assessment”, referred to the extent to which the local ecosystem for AHA is developed, integrated and established, joint projects ^[7–14,15,16,17,18]^, meetings ^[3,11,19–22]^, scaling up activities ^[23]^, conferences and workshops (www.whinn.dk and https://syddansksundhedsinnovationeipaha.wordpress.com/news/ and http://www.southdenmark.be/media/1701/dacob-upscaling-workshops-introduktion.pdf) and study-visits (http://www.syddansksundhedsinnovation.dk/service-menu/aktuelt/2016/juli-dec2016/500-gaester-fra-20-forskellige-lande-besoegte-syddansk-sundhedsinnovation-i-2016/) collaboration has allowed RS to share good practices and build cross border activities in a way that maximizes outcomes and reduces risks associated to investing in innovation to deliver a holistic approach to the achievement of the EIP on AHA objectives.

Revised criteria were introduced in 2016 to define and evaluate the RS (http://ec.europa.eu/research/innovation-union/pdf/active-healthy-ageing/2016_call_rs.pdf). One of the cornerstones within this was the requirement for the RS to build, and demonstrate that cross sectoral coalitions/alliances/partnerships have been implemented at regional level which support the research, development and adoption of new solutions and enable the scaling up and transferability of good practices within their region.

Seventy four RSs have been recognised in 2016 for their commitment to excellence in investing and scaling up innovative digital solutions for AHA (Table 1). Together the 74 RS s have committed to invest over € 4 billion in the next three years in the deployment and scaling up of digital innovation for AHA. This investment is expected to benefit over 5 million people in the next 3 years. It will also lay the foundations for a scalable EU market for digital innovation services and products meeting the needs of Europe’s ageing population and their carers.

Cumulatively, the RS commit to invest in specific main areas of digital innovation:

- Health promotion through personalised coaching and citizens empowerment;
- Disease prevention through big data and risk stratification;
- Digitally-enabled platforms for chronic disease management;
- Tools for integrating hospital care and community/social care;
- Programmes for upgrading tele-health and tele-care solutions to support independent living and quality of life of the ageing population;
- Multidisciplinary education, training and life-long learning innovative programs.

## III. REFERENCE SITE COLLABORATIVE NETWORK

It is beneficial to contribute to a continuous and constructive dialogue among the RS. Such a dialogue takes place participating in a collaborative network on an equal basis, regardless of their political and administrative structure. The Reference Site Collaborative Network (RSCN) brings together all EIP on AHA regions given RS status by the EC, and Candidate RS into a single forum.

### A. Vision

The RSCN aspires to be continually recognised by the EC as the principal forum and authority representing all EIP on AHA RS, and to establish connections with and across the Actions Groups (AG) in order to promote AHA. Our vision is to help our members accelerate the development, deployment and adoption of innovative health and social care solutions, proven AHA delivery models and digital solutions that provide real impact and contribute towards sustainability of services.

### B. Strategy

The overarching goals of the RSCN are to promote cooperation, share and transfer good practice in the implementationand scaling up of health and care strategies, policies and service delivery models. More specifically the RSCN will:

- Facilitate members to develop, share and adopt good practice and innovative solutions and technologies at scale;
- Influence and provide strategic input to bodies such as the EC, WHO, building on the knowledge and expertise of our regional members;
- Provide thought leadership through expert working groups;
- Provide a range of advisory and management services to members.

### C. Governance

Reference Sites have elected an RSCN Executive Board (EB) composed 8 Strategic Members and 2 Full Members appointed by the General Assembly (GA). All RS are eligible to participate in the GA, and one vote is allocated to each RS when conducting business. The EB has appointed two co- Chairs, two Deputy Vice Chairs and a Treasurer (Table 2).

EB Members are elected for 3 years and may be re-elected for one additional term. No Member may serve more than 2 consecutive terms. The EB meets at least twice per year.

The EB determines the strategies and actions of the RSCN. It will identify specific thematic Working Groups aimed at producing common operational projects in support of the EIP on AHA objectives.

The Secretariat of the RSCN will inform RS of new policy and funding developments; co-ordination of twinning and knowledge sharing events; establishing and maintaining links with candidate RS and Regions, the EU institutions and other organisations supporting EIP on AHA. The Secretariat shall be agreed and appointed by the EB. The secretariat is currently based in Montpellier (MACVIA France go.rscn@outlook.com)

### D. Membership

There are 5 categories of RSCN members (Table 3).

All RS are full members of the RSCN and one of their representatives will be a voting member at the GA. This representative will sit on the RSCN GA and will act on behalf of all the stakeholder organisations within the RS and ensure their views are represented. They will also be responsible for disseminating communications from the RSCN within their RS.

## IV. CURRENT ACTIONS

### A. Transfer of innovation: Twinning support scheme

The 2016 Transfer of Innovation Twinning Support Scheme was a pilot scheme launched by the EC with the support of the ScaleAHA Team (http://www.scale-aha.eu/home.html) to support regional deployment of innovation by partners of the EIP on AHA through the reimbursement of expenses incurred in the transfer of innovative practices. Under this scheme, twenty pairs of RS (Table 4) have been provided with financial support for study visits between experts in the adopting and originator organisations. Twinning project represents an opportunity for both patients and healthcare professionals, cause it facilitate the assessment of impact of digitally enabled innovations in a uniform way.

**Figure 1:**
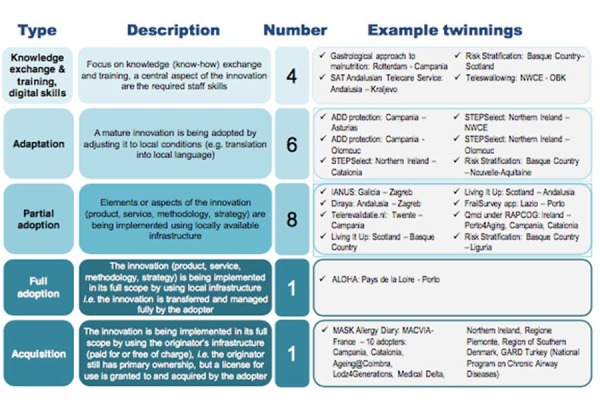
Twinning archetypes

MThe first results are promising, and the process should be further refined taking into consideration lessons learnt and recommendations by the pilot twinning organisations. The final report (http://www.scaleaha.eu/fileadmin/scaleaha/documents/scaleaha_d5.4_finalstudyreport.pdf) presents interim results of the twinning activities, which include discussions about barriers and challenges faced, success factors leveraged, plans and strategies on moving forward, and recommendations for the future. It also presented twinning archetypes (Figure 1).

The ScaleAHA team also provided a number of recommendations coming from the RS and the twinning activities for policy makers, and for better organisation of future initiatives such as a second call for transfer of innovation. Other recommendations concerned future calls for RS, funding utilization support and the assessment of impact of digitally enabled innovations in a uniform way.

### B. Interactions with the Commission

The RSCN is registered at the EC Transparency Register (ID: 583454420450-89) since January 2016. The Transparency Register has been set up to answer core questions such as what interests are being pursued, by whom and with what budgets. The system is operated jointly by the European Parliament and the EC. The RSCN is a member of the eHealth Stakeholder Group (eHSG), set up by DG SANTE and DG CNECT through a call for expression of interest in January 2016. Currently Andalusia (representing RSCN as its vice-chair) is rapporteur for the working group on Care Continuum within the eHSG. RSCN is responding to public consultations and contributing to the decision-making process at the EC level.

### C. Interactions with the CSA

The WE4AHA Coordination and Support Action (CSA is aimed at advancing the effective, large-scale uptake and impact of Digital Innovation for AHA, mobilizing relevant stakeholders to help develop and implement three EU guided activities: Innovation 2 Market, Blueprint on Digital Transformation of Health and Care for the Ageing Society, and EIP on AHA. Hence, the RSCN has a bidirectional connection with the CSA: is supported by the CSA for some specific horizontal activities and ensures that the EU guided activities are developed by taking advantage of the contributions of all partners of the EIP on AHA.

Within the WE4AHA CSA, the RSCN will be responsible for some actions:

1. Twinning programs for large scale-up digital solutions;
2. Organize at least 6 thematic workshops including:
  - Health Tourism Brussels, 27 February 2018): leader: PROMIS,
  - POLLAR (CoP 2019),
  - Thematic workshops in collaboration with EUREGHA,
  - A call will be opened each year to obtain topics and locations from RS members. Part of funding will be available for these events;
3. Support for event of regional stakeholders to be replicated across the EU;
4. Help to launch the next call for RS;
5. Identification of the key elements to map the quadruple helix ecosystem;.
6. Evaluation of RS progress;
7. Release content for dissemination activities.

### D. Interactions with the other EU Organisations

The RSCN recognizes the benefit to be achieved from working closely with other EU networks and partners, particularly those whose aims, and goals overlap with its own. Nick Batey (RSCN) connects the work of the RSCN with that of EUREGHA. The RSCN also works with the ECHAlliance as part of the Coalition of the Willing (CoW).

### E. Current RSCN involvement in EC projects

The RSCN is currently (December 2018) involved in three European Projects. VIGOUR, a 3rd Health Programme project, seeks to support care authorities in progressing the transformation of their health and care systems to provide sustainable models for integrated care. DigitalHealthEurope, a H2020 CSA project, will provide comprehensive, centralized support to the digital transformation of health and care (DTHC) priorities of the Digital Single Market. The project will support large-scale deployment of digital solutions for person-centered integrated care. EURIPHI, also a H2020 CSA project, has as its vision to build out around the Most Economic Advantageous Tender (MEAT) Value Based Procurement framework which will be made accessible with adaptions necessary to support the cross-border PPI leading to “MEAT Value Based PPI”.

The inclusion of the RSCN in these projects highlights the strategic position of the organisation within the consortiums, acting as a catalyst to foster scaling-up across regions and countries. With the RSCN, the projects have first-hand access not only to regions which are innovation leaders, but also to regions who are less mature in their person-centred integrated care. The RSCN closely support tasks related to identifying best practices by helping to assess the characteristics and the impact of the innovative approach. It will facilitate partnerships with other regions for the updating of existing guidance material, the conduction of twinning activities and wider scaling-up guidance.

The RSCN is aiming at answering to the need for a collaborative approach to facilitate joint reflection and action in sharing and transferring best practices in the development and scaling up of health and care strategies, policies and service delivery models.

## IV. SOME EXAMPLES OF RSCN ACHIEVEMENTS

### A. Programma Mattone Internazionale Salute

Established in 2013 as a project of the MoH in 2016, Programma Mattone Internazionale Salute (ProMIS) became an institutional structure aimed at creating a permanent dialogue and synergies among Italian Regions, as well as with the EU health policies and systems. ProMIS provides opportunities of information and discussion, organizing workshops and conferences, satisfying the needs jointly expressed by Italian regions. It also disseminates European calls, stimulating the participation of Italian clusters to the consortia and supporting the regions in the coordination for the participation to the calls. The program has developed preparatory activities to support the Regions in their application to become a RS and to submit commitments, explaining the details of the calls, facilitating the access to the useful information in order to prepare the proposal, thus making the Italian Regions collaborate at their best with the other European Regions ^[24]^. Among the 74 RSs awarded by the EC in 2016, 11 are Italian Regions that have been assigned one or more stars according to the maturity: Campania, Emilia Romagna, Friuli Venezia Giulia, Lazio, Liguria, Lombardy, Piedmont, Puglia, Tuscany, Veneto and the Autonomous Province of Trento. In order to define a common RS “model” and give Regions a structure to assess the effectiveness/validity of their strategies, Italian Regions agreed to draft a document ***“Methodology for the Italian Reference Sites: Which organizational structure?”*** where RS management, methods and tools are described, supported by validation elements of the RS model at European level. Every year the updated version of “EIP-AHA Italy: the Italian experience in the framework of the European Innovation Partnership on Active and Healthy Ageing” is also published, which is the focal document describing Italian RS activity and all the relevant European and national initiatives linked with EIP-AHA.

### B. Global Alliance Chronic Respiratory Diseases Regional Network (Turkey)

The Global Alliance Chronic Respiratory Diseases Regional Network is the National Control Program of Turkish MoH on chronic airway disease with 64 collaborating parties which can be used as a model for EIP on AHA RS ^[25,26]^.

### C. Coimbra activities of the RSCN

Instituto Pedro Nunes, a member of Ageing@Coimbra reference site, was the local organizer of the Ambient Assisted Living Forum 2017 (2–4 October, 2017) ^[27]^. The program of the meeting included the workshop “Bridges between Europe – integrating health and social care towards innovation” with representatives of RSCN (Maddalena Illario and João Malva). The RS Ageing@Coimbra has been leader of the innovative activity joining senior citizens and innovators in the Forum. From local Third Age Universities and nursing homes, 120 +65 people have been invited to visit the technological exhibitors in the Forum and to perform the evaluation of the technologies. At the end of this exercise, the most favorite technologies were ranked and a winner was selected. All the exposed technologies received an assessment report, including recommendations provided by the end-users ^[28]^.

### D. Mobile Airways Sentinel network (MASK@Twinning)

The aim of MASK@Twinning is to transfer innovation from an App developed by the MACVIA-France (MASK, TLR9) ^[29–35]^ to other RSs ^[36]^. MASK follows the criteria for Good Practices of the CHRODIS Joint Action ^[37]^ and its privacy is in line with the Article 28 EU General Data Protection Regulation (EU-GDPR) ^[38]^. The phenotypic characteristics of rhinitis and asthma multimorbidity ^[39]^ in adults and the elderly are compared using validated information and communication technology (ICT) tools (i.e. the Allergy Diary and CARAT: Control of Allergic Rhinitis and Asthma Test) in 29 RSs, regions or countries across Europe and beyond. This will improve understanding, assessment of burden, diagnosis and management of rhinitis in the elderly by comparison with an adult population. Specific objectives are to: (i) assess the percentage of adults and elderly who are able to use the Allergy Diary, (ii) study phenotypic characteristics and treatment over a period of one year of rhinitis and asthma multimorbidity at baseline (cross-sectional study) and (iii) follow-up using visual analogue scale (VAS). This part of the study may provide some insight into the differences between the elderly and adults in terms of response to treatment and practice as well as precision medicine ^[40]^. Finally (iv) work productivity is examined in adults. The first results of MASK@Twinning are very promising and over 400 patients have been recruited. A pilot study showed that the questionnaire for physicians (EUFOREA-ARIA website, www.euforea.eu/) ^[40]^ is appropriate. This project also allowed MASK to be deployed in the entire country with the national society in France and Germany. Moreover, MASK@Twinning has been endorsed by the European Academy of Allergy and Clinical Immunology (EAACI), the European Respiratory Society (ERS), the International Primary Care Airways Group (IPCRG), two major European patients’ organisations (EFA, European Federation of Allergy and Airways Diseases Patients’ Associations and ELF, European Lung Foundation) and an international patient’s organization (GAAP) ^[41]^. It is WHO Global Alliance against Chronic Respiratory Diseases (GARD) demonstration project. MASK@Twinning centers have been included in a 2018 EIT Health Innovation-by-Design project (POLLAR: Impact of Air Pollution in Asthma and Rhinitis).

### E. Participation of RSCN to International Projects

RSCN co-sponsored a WHO-GARD meeting in Brussels on the impact of air pollution in chronic respiratory diseases (10^th^ November, 2018). It is also co-sponsoring an EU Summit held by the Minister of Health of Lithuania on the management of chronic respiratory diseases (Vilnius, 23^rd^ March, 2018) and the consensus meeting on self-management in airways diseases (EIT Health, WHO GARD), December 2018.

## V. RSCN CHANGE MANAGEMENT MODEL

The RSCN follows a change management strategy to accomplish its vision and mission. Although theories may seem abstract and impractical, they can help to solve common problems ^[42]^. The 3-Step model of the Lewin’s approach ^[43]^ dominated the change management theory and practice for over 50 years. Although criticized, it is still used ^[44,45]^ and has great interest in its simplicity ^[46]^. The model posits the 3-step sequence of change: unfreezing, moving, and refreezing ^[45,47]^. Kotter ^[48]^ has added to the collective change knowledge to expand upon Lewin’s original Theory (Table 5) ^[43]^.

Many different projects have shown the importance of the EIP on AHA to achieve its goals. It is, however, urgent that the concept of AHA is more widely and rapidly translated into practice. The RSCN is one of the key tools of the EIP on AHA to transfer concepts to practice (Step1).

The 74 RS of the EIP on AHA represent an exceptional group committed to the deployment of AHA in EU regions and beyond. The RSCN represents a guiding coalition lead by its executive board and strategic members (Step2).

The RSCN vision and strategy are clearly defined (Step 3).

The change vision is disseminated through a dedicated website and using all means for communication. This paper is an important communication tool. A newsletter will be regularly published (Step 4).

Organizational processes and structures are in place and an ASBL is set and will help to remove the obstacles involved in the process of change. The Regional Events will help to empower others (Step 5).

Short term wins have already been obtained (see chapter 4) and a strategy for next year is in place (Step 6).

The goals of step 7 ^[48]^ are to achieve continuous improvement by analysing the success stories individually and improving from those individual experiences.

The goals of step 8 ^[48]^ are:

1. Discuss the successful stories related to change initiatives widely.
2. Ensure that the change becomes an integral part of the practice and is highly visible.
3. Ensure that the support of the existing as well as the new leaders continues to extend towards the change.

## 











## Figures and Tables

**Table 1:** List and contacts of Reference Sites

	Reference Site	Country	Main Contact Point	Second Contact point or Coordinator
1	Amsterdam Metropolitan Area	The Netherlands	Sabina van der Veen	
2	Andalusia	Spain	Ana Carriazo	Mercedes Garcia
3	Aragon	Spain	Juan I. Coll Clavero	
4	Arsenàl.IT - Veneto’s Research Centre for eHealth Innovation	Italy	Claudio Saccavini	
5	Asturias	Spain	Nerea Eguren	Ana Bernardo Suarèz
6	Aust-Agder County and Vest-Agder County	Norway	Rune Fensli	
7	Baden-Württemberg	Germany	Daniel Buhr	
8	Balearic Islands	Spain	Elena Cabeza	
9	Barcelona Province	Spain	Alejandra Millet Pi-Figueras	
10	Basque Country	Spain	Jon Darpón Sierra	Esteban de Manuel Keenoy
11	Campania	Italy	Maddalena Illario	
12	Catalonia	Spain	Antoni Dedeu Baraldes	
13	Centro	Portugal	Joao Malva	Ana Abrunhosa
14	City of Augsburg	Germany	Andreas W. Huber	
15	City of Badalona	Spain	Jordi Piera Jiménez	
16	City of Helsinki	Finland	Sanna Hartman	Mr. Heikki Kallasvaara Arja Peiponen
17	City of Kraljevo	Serbia	Milan Vukovic	
18	City of Kuopio	Finland	Markku Tervahauta	
19	City of Liverpool	United Kingdom	Dave Horsfield	
20	City of Oulu	Finland	Anne Rännäli-Kontturi	Salla Hirvonen
21	City of Sofia	Bulgaria	Yanko Kuzmanov	Stoicho Katsarov
22	City of Terrassa	Spain	Manel Balcells Diaz	
23	City of Zagreb	Croatia	Antonija Balenović	
24	East of France	France	Valentin Ledroit	
25	Emilia-Romagna	Italy	Maria Luisa Moro	Papini Donato Brigida Marta
26	Federal Ministry for Family Affairs, Senior Citizens, Women and Youth	Germany	Peter Kupferschmid	
27	Flanders	Belgium	Loes Houthuys	
28	Friuli Venezia Giulia	Italy	Arrigo Venchiarutti	
29	Galicia	Spain	Susana Fernandez Nocelo	
30	Global Alliance Chronic Respiratory Diseases Regional Network	Turkey	Arzu Yorgancıoglu	
31	Greater Manchester	United Kingdom	Amanda Risino	Paul Mc Garry
32	Healthy Ageing Network Northern Netherlands	The Netherlands	Daan Bultje	
33	Heraklion-Crete	Greece	Panayiotis Mitsias	
34	Île-de-France	France	Louis Potel	
35	Kiev-Zhitomir	Ukraine	Leonid Bogatyrchuk	
36	Kinzigtal	Germany	Dirk Günther	
37	Lazio	Italy	Ursula Kirchmayer	
38	Liguria	Italy	Lorenzo Bertorello	
39	Limburg	Belgium	Laura Visconti	
40	Lodz Province	Poland	Lucyna A. Wozniak	
41	Lombardy	Italy	Maurizio Bersani	
42	MACVIA France Network	France	Jean Bousquet	
43	Madrid	Spain	Teresa Chavarria Giménez	
44	Medical Delta	The Netherlands	Agaath Sluijter	
45	Metropolitan Area of Porto (Porto4Agein g)	Portugal	Elísio Costa	
46	Milan Metropolitan-Bergamo Province	Italy	Maria Romano	
47	Region de Murcia	Spain	Beatriz Martínez-Lozano Aranaga	
48	Norrbotten	Sweden	Lisa Lundgren	
49	North Brabant Province	The Netherlands	Peter Portheine	
50	North East England	United Kingdom	Graham Armitage	
51	North West Coast of England	United Kingdom	Phil Jennings	Eleanor Garnett-Bentley Andrew Cooper
52	Northern Ireland	United Kingdom	Elaine Colgan	
53	Nouvelle-Aquitaine	France	Carole Doucet	
54	Oberbergische r Kreis	Germany	Wolfgang Goetzke	Judith Brehm
55	Olomouc	Czech Republic	Zdenek Gütter	
56	Pays De La Loire	France	Hajjam Jawad	
57	Piedmont	Italy	Valeria Romano	
58	Pirkanmaa	Finland	Anja Tuulonen	
70	Twente	The Netherlands	Miriam Vollenbroek-Hutten	
71	Valencian Community	Spain	Charo Penadés	Javier Gamez
72	Wales	United Kingdom	Nick Batey	
73	West Flanders Province	Belgium	Inge Taillieu	
74	Yorkshire and the Humber	United Kingdom	Stephen Stericker	
75	Zealand	Denmark	Esther Bülow Davidsen	

**Table 2:** RSCN Executive Baord

Co-chairs	J Bousquet (MACVIA-France), M Illario (Campania)
Vice-Chairs	N Batey (Wales), A Carriazo (Andalucia)
Treasurer	J Malva (Ageing@Coimbra)
Scientific adviser	N Guldemond (Delta Medica, NL)
Members	E Colgan (Northern Ireland), J Hajjam (Pays de la Loire), M Perälä-Heape (Oulu, Finland)
Adviser	J Farrell

**Table 3:** RSCN Membership categories

	Membership	Description	Paying fee^*^	Participation in GA, WG, conference	Voting right at GA	Ex Board member
1	Full member	Full membership is open to all RS approved by the European Commission	No	Yes	Yes	Yes (up to 2)
2	Strategic member	RS that take active and leading roles in the network	In species	Yes	Yes	Yes (but max 10)
3	Honorary member	Individuals distinguished in the fields of AHA They are appointed by the GA upon proposal from the Executive Board	No	Yes	No	No
4	Affiliate member	Organisations not part of an existing RS but with an interest in pursuing similar goals Only legal entities duly constituted in accordance with the laws of their country of origin, can become an associate member They are appointed by the GA upon proposal from the Executive Board	In species	Yes	No	No
5	Observer	Individuals with an interest in AHA who may contribute to the work of the RSCN They are appointed by the GA upon proposal from the Executive Board Individuals working for lobbying groups or for organisations with a commercial purpose will not be accepted as observers.	No	Can only participate, in an advisory capacity in the GA, the WG and the conferences upon invitation by the Chair.	No	No

**Table 4:** List of Twinnings

	Originator 2016 RS name	Adopter(s) 2016 RS name	Contact person
1	MACVIA-France Network (FR)	Andalucia Aragon Campania Catalonia City of Helsinki Coimbra Heraklion Kohln-Bohn Region Life Tech Valley Liguria Lodz Medical Delta Milan Metropolitan - Bergamo Province NHS 24 Northern Ireland Olomouc Pays de la Loire Porto Puglia Regione Piemonte Regione Toscany Region of Southern Denmark Turkey (Global Alliance Chronic ARIA Sweden ARIA Lithuania ARIA Argentina ARIA Australia ARIA Brazil ARIA Mexico	Jean Bousquet, MACVIA jean.bousquet@orange.fr
2	Northern Ireland (UK)	Catalonia (ES)	Michael Scott, Northern Ireland (UK) email: DrMichael.Scott@northerntrust.hscni.net
3	Northern Ireland (UK)	Olomouc (CZ)	Michael Scott, Northern Ireland (UK) email:
4	Pays de la Loire (FR)	Porto Metropolitan Area - Porto4Ageing (PT)	Elísio Costa, Porto Metropolitan Area emcosta@ff.up.pt
5	Northern Ireland (UK)	North West Coast of England (UK)	Michael Scott, Northern Ireland (UK) email: DrMichael.Scott@northerntrust.hscni.net
6	Campania (IT)	Asturias (ES)	Ángel Retamar Arias, Asturias (ES) email:
7	Lazio (IT)	Porto Metropolitan Area - Porto4Ageing (PT)	Elísio Costa, Porto Metropolitan Area emcosta@ff.up.pt
8	Twente (NL)	Campania (IT)	Lex van Velsen email:
9	Andalusia (ES)	City of Zagreb (HR)	Ana Carriazo anam.carriazo@juntadeandalucia.es
10	Basque Country (ES)	Nouvelle-Aquitaine (FR)	Carole Doucet, Nouvelle-Aquitaine email: carole.doucet@nouvelle-aquitaine.fr
11	Medical Delta Rotterdam (NL)	Campania (IT)	Edwig Goossens email:
12	Republic of Ireland Regional Network (COLLAGE)	Campania (IT) Catalonia (ES) Metropolitan Area of Porto (Porto4Ageing)	Rónán O’Caoimh (COLLAGE) ronan.ocaoimh@nuigalway.ie
13	Basque Country (ES)	Liguria (IT)	Dolores Verdoy, Basque Country (ES) dverdoy@kronikgune.org
14	Galicia (ES)	City of Zagreb (BG)	Susana Fernández Nocelo susana.fernandez.nocelo@sergas.es
15	Scotland (UK)	Basque Country (ES)	Dolores Verdoy, Basque Country (ES) dverdoy@kronikgune.org
16	Campania (IT)	Olomouc (CZ)	Zdenek Gütter, Olomouc (CZ) gutter@ntmc.cz
17	Basque Country (ES)	Scotland (UK)	Donna Henderson, Scotland (UK) donna.henderson1@nhs.net
18	North West Coast of England (UK)	Oberbergischer Kreis (DE)	Wolfgang Goetzke, Oberbergischer Kreis (DE) info@health-region.de
19	Scotland (UK)	Andalusia (ES)	Ana Carriazo, Andalousia (ES) anam.carriazo@juntadeandalucia.es
20	Andalusia (ES)	City of Kraljevo (SRB)	Milan Vukovic, City of Kraljevo (SRB) milan.vukovic@belit.co.rs

**Table 5:** The Kotter’s model of change management Adapted from ^[45]^

Lewin	Kotter
**Unfreezing**	Step 1: Establish a sense of urgency
	Step 2: Create a guiding coalition
	Step 3: Develop a vision and strategy
**Moving**	Step 4: Communicate the change vision
	Step 5: Empower others to act on the vision
	Step 6: Generate short-term wins
	Step 7: Consolidate gains and produce more change
**Refreezing**	Step 8: Anchor new approaches in the culture and institutionalize the changes

## Abbreviations

AHA: active and healthy ageing
EIP: European Innovation Partnership
EIP on AHA: European Innovation Partnership on Active and Healthy Ageing
EU: European Union
EC: European Commission
RS: Reference Site
RSCN: Reference Site Collaborative Network
