# Supplementary material for: The Reference Site Collaborative Network of the European Innovation Partnership on Active and Healthy Ageing
Source: Transl Med UniSa. 2019 Jan 6;19:66–81. (PMC6581486)
Supplement: Supplementary file 1 [file TM-19-066-s001.doc]

|  | **Reference Site** | **Country** | **Main Contact Point** | **Second Contact point or Coordinator** |
| --- | --- | --- | --- | --- |
| 1 | Amsterdam Metropolitan Area | The Netherlands | Sabina van der Veen |  |
| 2 | Andalusia | Spain | Ana Carriazo | Mercedes Garcia |
| 3 | Aragon | Spain | Juan I. Coll Clavero |  |
| 4 | Arsenàl.IT - Veneto's Research Centre for eHealth Innovation | Italy | Claudio Saccavini |  |
| 5 | Asturias | Spain | Nerea Eguren | Ana Bernardo Suarèz |
| 6 | Aust-Agder County and Vest-Agder County | Norway | Rune Fensli |  |
| 7 | Baden-Württemberg | Germany | Daniel Buhr |  |
| 8 | Balearic Islands | Spain | Elena Cabeza |  |
| 9 | Barcelona Province | Spain | Alejandra Millet Pi-Figueras |  |
| 10 | Basque Country | Spain | Jon Darpón Sierra | Esteban de Manuel Keenoy |
| 11 | Campania | Italy | Maddalena Illario |  |
| 12 | Catalonia | Spain | Antoni Dedeu Baraldes |  |
| 13 | Centro | Portugal | Joao Malva | Ana Abrunhosa |
| 14 | City of Augsburg | Germany | Andreas W. Huber |  |
| 15 | City of Badalona | Spain | Jordi Piera Jiménez |  |
| 16 | City of Helsinki | Finland | Sanna Hartman | Mr. Heikki Kallasvaara  Arja Peiponen |
| 17 | City of Kraljevo | Serbia | Milan Vukovic |  |
| 18 | City of Kuopio | Finland | Markku Tervahauta |  |
| 19 | City of Liverpool | United Kingdom | Dave Horsfield |  |
| 20 | City of Oulu | Finland | Anne Rännäli-Kontturi | Salla Hirvonen |
| 21 | City of Sofia | Bulgaria | Yanko Kuzmanov | Stoicho Katsarov |
| 22 | City of Terrassa | Spain | Manel Balcells Diaz |  |
| 23 | City of Zagreb | Croatia | Antonija Balenović |  |
| 24 | East of France | France | Valentin Ledroit |  |
| 25 | Emilia-Romagna | Italy | Maria Luisa Moro | Papini Donato  Brigida Marta |
| 26 | Federal Ministry for Family Affairs, Senior Citizens, Women and Youth | Germany | Peter Kupferschmid |  |
| 27 | Flanders | Belgium | Loes Houthuys |  |
| 28 | Friuli Venezia Giulia | Italy | Arrigo Venchiarutti |  |
| 29 | Galicia | Spain | Susana Fernandez Nocelo |  |
| 30 | Global Alliance Chronic Respiratory Diseases Regional Network | Turkey | Arzu Yorgancıoglu |  |
| 31 | Greater Manchester | United Kingdom | Amanda Risino | Paul Mc Garry |
| 32 | Healthy Ageing Network Northern Netherlands | The Netherlands | Daan Bultje |  |
| 33 | Heraklion-Crete | Greece | Panayiotis Mitsias |  |
| 34 | Île-de-France | France | Louis Potel |  |
| 35 | Kiev-Zhitomir | Ukraine | Leonid Bogatyrchuk |  |
| 36 | Kinzigtal | Germany | Dirk Günther |  |
| 37 | Lazio | Italy | Ursula Kirchmayer |  |
| 38 | Liguria | Italy | Lorenzo Bertorello |  |
| 39 | Limburg | Belgium | Laura Visconti |  |
| 40 | Lodz Province | Poland | Lucyna A. Wozniak |  |
| 41 | Lombardy | Italy | Maurizio Bersani |  |
| 42 | MACVIA France Network | France | Jean Bousquet |  |
| 43 | Madrid | Spain | Teresa Chavarria Giménez |  |
| 44 | Medical Delta | The Netherlands | Agaath Sluijter |  |
| 45 | Metropolitan Area of Porto (Porto4Ageing) | Portugal | Elísio Costa |  |
| 46 | Milan Metropolitan - Bergamo Province | Italy | Maria Romano |  |
| 47 | Region de Murcia | Spain | Beatriz Martínez-Lozano Aranaga |  |
| 48 | Norrbotten | Sweden | Lisa Lundgren |  |
| 49 | North Brabant Province | The Netherlands | Peter Portheine |  |
| 50 | North East England | United Kingdom | Graham Armitage |  |
| 51 | North West Coast of England | United Kingdom | Phil Jennings | Eleanor Garnett-Bentley  Andrew Cooper |
| 52 | Northern Ireland | United Kingdom | Elaine Colgan |  |
| 53 | Nouvelle-Aquitaine | France | Carole Doucet |  |
| 54 | Oberbergischer Kreis | Germany | Wolfgang Goetzke | Judith Brehm |
| 55 | Olomouc | Czech Republic | Zdenek Gütter |  |
| 56 | Pays De La Loire | France | Hajjam Jawad |  |
| 57 | Piedmont | Italy | Valeria Romano |  |
| 58 | Pirkanmaa | Finland | Anja Tuulonen |  |
| 70 | Twente | The Netherlands | Miriam Vollenbroek-Hutten |  |
| 71 | Valencian Community | Spain | Charo Penadés | Javier Gamez |
| 72 | Wales | United Kingdom | Nick Batey |  |
| 73 | West Flanders Province | Belgium | Inge Taillieu |  |
| 74 | Yorkshire and the Humber | United Kingdom | Stephen Stericker |  |
| 75 | Zealand | Denmark | Esther Bülow Davidsen |  |

Table 1. List and contacts of Reference Sites
