# Supplementary material for: The Reference Site Collaborative Network of the European Innovation Partnership on Active and Healthy Ageing
Source: Transl Med UniSa. 2019 Jan 6;19:66–81. (PMC6581486)
Supplement: Supplementary file 2 [file TM-19-066-s002.doc]

| Co-chairs | J Bousquet (MACVIA-France), M Illario (Campania) |
| --- | --- |
| Vice-Chairs | N Batey (Wales), A Carriazo (Andalucia) |
| Treasurer | J Malva (Ageing@Coimbra) |
| Scientific adviser | N Guldemond (Delta Medica, NL) |
| Members | E Colgan (Northern Ireland), J Hajjam (Pays de la Loire), M Perälä-Heape (Oulu, Finland) |
| Adviser | J Farrell |

Table 1. RSCN Executive Baord
