# Supplementary material for: The Reference Site Collaborative Network of the European Innovation Partnership on Active and Healthy Ageing
Source: Transl Med UniSa. 2019 Jan 6;19:66–81. (PMC6581486)
Supplement: Supplementary file 3 [file TM-19-066-s003.doc]

|  | **Membership** | **Description** | **Paying fee*** | **Participation in GA, WG, conference** | **Voting right at GA** | **Ex Board member** |
| --- | --- | --- | --- | --- | --- | --- |
| 1 | Full member | Full membership is open to all RS approved by the European Commission | No | Yes | Yes | Yes (up to 2) |
| 2 | Strategic member | RS that take active and leading roles in the network | In species | Yes | Yes | Yes (but max 10) |
| 3 | Honorary member | - Individuals distinguished in the fields of AHA - They are appointed by the GA upon proposal from the Executive Board | No | Yes | No | No |
| 4 | Affiliate member | - Organisations not part of an existing RS but with an interest in pursuing similar goals - Only legal entities duly constituted in accordance with the laws of their country of origin, can become an associate member - They are appointed by the GA upon proposal from the Executive Board | In species | Yes | No | No |
| 5 | Observer | - Individuals with an interest in AHA who may contribute to the work of the RSCN - They are appointed by the GA upon proposal from the Executive Board - Individuals working for lobbying groups or for organisations with a commercial purpose will not be accepted as observers. | No | Can only participate, in an advisory capacity in the GA, the WG and the conferences upon invitation by the Chair. | No | No |

Table 1. RSCN Membership categories
