# Supplementary material for: The Reference Site Collaborative Network of the European Innovation Partnership on Active and Healthy Ageing
Source: Transl Med UniSa. 2019 Jan 6;19:66–81. (PMC6581486)
Supplement: Supplementary file 4 [file TM-19-066-s004.doc]

|  | **Originator 2016 RS name** | **Adopter(s) 2016 RS name** | **Contact person** |
| --- | --- | --- | --- |
| 1 | MACVIA-France Network (FR) | 1. Andalucia 2. Aragon 3. Campania 4. Catalonia 5. City of Helsinki 6. Coimbra 7. Heraklion 8. Kohln-Bohn Region 9. Life Tech Valley 10. Liguria 11. Lodz 12. Medical Delta 13. Milan Metropolitan - Bergamo Province 14. NHS 24 15. Northern Ireland 16. Olomouc 17. Pays de la Loire 18. Porto 19. Puglia 20. Regione Piemonte 21. Regione Toscany 22. Region of Southern Denmark 23. Turkey (Global Alliance Chronic 24. ARIA Sweden 25. ARIA Lithuania 26. ARIA Argentina 27. ARIA Australia 28. ARIA Brazil 29. ARIA Mexico | Jean Bousquet, MACVIA  [jean.bousquet@orange.fr](mailto:jean.bousquet@orange.fr) |
| 2 | Northern Ireland (UK) | Catalonia (ES) | Michael Scott, Northern Ireland (UK) email: DrMichael.Scott@northerntrust.hscni.net |
| 3 | Northern Ireland (UK) | Olomouc (CZ) | Michael Scott, Northern Ireland (UK) email: |
| 4 | Pays de la Loire (FR) | Porto Metropolitan Area - Porto4Ageing (PT) | Elísio Costa, Porto Metropolitan Area  [emcosta@ff.up.pt](mailto:emcosta@ff.up.pt) |
| 5 | Northern Ireland (UK) | North West Coast of England (UK) | Michael Scott, Northern Ireland (UK) email: DrMichael.Scott@northerntrust.hscni.net |
| 6 | Campania (IT) | Asturias (ES) | Ángel Retamar Arias, Asturias (ES) email: |
| 7 | Lazio (IT) | Porto Metropolitan Area - Porto4Ageing (PT) | Elísio Costa, Porto Metropolitan Area [emcosta@ff.up.pt](mailto:emcosta@ff.up.pt) |
| 8 | Twente (NL) | Campania (IT) | Lex van Velsen email: |
| 9 | Andalusia (ES) | City of Zagreb (HR) | Ana Carriazo [anam.carriazo@juntadeandalucia.es](mailto:anam.carriazo@juntadeandalucia.es) |
| 10 | Basque Country (ES) | Nouvelle-Aquitaine (FR) | Carole Doucet, Nouvelle-Aquitaine email: [carole.doucet@nouvelle-aquitaine.fr](mailto:carole.doucet@nouvelle-aquitaine.fr) |
| 11 | Medical Delta Rotterdam (NL) | Campania (IT) | Edwig Goossens email: |
| 12 | Republic of Ireland Regional Network (COLLAGE) | Campania (IT) Catalonia (ES) Metropolitan Area of Porto (Porto4Ageing) | Rónán O'Caoimh (COLLAGE) [ronan.ocaoimh@nuigalway.ie](mailto:ronan.ocaoimh@nuigalway.ie) |
| 13 | Basque Country (ES) | Liguria (IT) | Dolores Verdoy, Basque Country (ES) [dverdoy@kronikgune.org](mailto:dverdoy@kronikgune.org) |
| 14 | Galicia (ES) | City of Zagreb (BG) | Susana Fernández Nocelo [susana.fernandez.nocelo@sergas.es](mailto:susana.fernandez.nocelo@sergas.es) |
| 15 | Scotland (UK) | Basque Country (ES) | Dolores Verdoy, Basque Country (ES) [dverdoy@kronikgune.org](mailto:dverdoy@kronikgune.org) |
| 16 | Campania (IT) | Olomouc (CZ) | Zdenek Gütter, Olomouc (CZ) [gutter@ntmc.cz](mailto:gutter@ntmc.cz) |
| 17 | Basque Country (ES) | Scotland (UK) | Donna Henderson, Scotland (UK) [donna.henderson1@nhs.net](mailto:donna.henderson1@nhs.net) |
| 18 | North West Coast of England (UK) | Oberbergischer Kreis (DE) | Wolfgang Goetzke, Oberbergischer Kreis (DE) [info@health-region.de](mailto:info@health-region.de) |
| 19 | Scotland (UK) | Andalusia (ES) | Ana Carriazo, Andalousia (ES) [anam.carriazo@juntadeandalucia.es](mailto:anam.carriazo@juntadeandalucia.es) |
| 20 | Andalusia (ES) | City of Kraljevo (SRB) | Milan Vukovic, City of Kraljevo (SRB) [milan.vukovic@belit.co.rs](mailto:milan.vukovic@belit.co.rs) |

Table 1. List of Twinnings
