# Supplementary material for: The Reference Site Collaborative Network of the European Innovation Partnership on Active and Healthy Ageing
Source: Transl Med UniSa. 2019 Jan 6;19:66–81. (PMC6581486)
Supplement: Supplementary file 5 [file TM-19-066-s005.doc]

| **Lewin** | **Kotter** |
| --- | --- |
| **Unfreezing** | Step 1: Establish a sense of urgency |
|  |  |
|  | Step 2: Create a guiding coalition |
|  | Step 3: Develop a vision and strategy |
| **Moving** | Step 4: Communicate the change vision |
|  | Step 5: Empower others to act on the vision |
|  | Step 6: Generate short-term wins |
|  | Step 7: Consolidate gains and produce more change |
| **Refreezing** | Step 8: Anchor new approaches in the culture and institutionalize the changes |

Table 1: The Kotter’s model of change management - Adapted from
